# Selective *in vitro* photothermal nano-therapy of MRSA infections mediated by IgG conjugated gold nanoparticles

**DOI:** 10.1038/srep39466

**Published:** 2016-12-23

**Authors:** Lucian Mocan, Cristian Matea, Flaviu A. Tabaran, Ofelia Mosteanu, Teodora Pop, Cosmin Puia, Lucia Agoston-Coldea, Diana Gonciar, Erszebet Kalman, Gabriela Zaharie, Cornel Iancu, Teodora Mocan

**Affiliations:** 1 Nanomedicine Department, Regional Institute of Gastroenterology and Hepatology Octavian Fodor Cluj, Romania; 2 Department of Surgery, University of Medicine and Pharmacy, “Iuliu Hatieganu”, Croitorilor 19-21, Cluj-Napoca, Romania; 3 Department of Pathology, University of Agricultural Sciences and Veterinary Medicine, Faculty of Veterinary Medicine 3-5 Manastur Street, 400372 Cluj-Napoca, Romania; 4 Department of Gastroenterology, University of Medicine and Pharmacy, “Iuliu Hatieganu”, Croitorilor 19-21, Cluj-Napoca, Romania; 5 Department of Internal Medicine, University of Medicine and Pharmacy, “Iuliu Hatieganu”, Clinicilor 3-5, Cluj-Napoca, Romania; 6 Department of Epidemiology, Regional Institute of Gastroenterology and Hepatology Octavian Fodor Cluj, Romania; 7 Department of Neonatology, “Iuliu Hatieganu” University of Medicine and Pharmacy, Cluj-Napoca, Romania; 8 Department of Physiology, University of Medicine and Pharmacy, “Iuliu Hatieganu”, Croitorilor 19-21, Cluj-Napoca, Romania

## Abstract

There are serious systemic infections associated with methicillin-resistant *Staphylococcus aureus* (MRSA) and several other types of bacteria leading to the deaths of millions of people globally. This type of mortality is generally caused by the increasing number of antibiotic-resistant organisms, a consequence of evolution via natural selection. After the synthesis of gold nanoparticles (GNPs) by wet chemistry, bio-functionalization with IgG molecules was performed. Following administration of IgG-GNPs to MRSA cultures at various concentrations and various incubation time laser irradiation was performed. To assess the selectivity and specificity of the proposed treatment the following methods were used: flow cytometry, contrast phase microscopy, and by fluorescence microscopy. The results in our study indicate that following administration of IgG-GNPs biomolecule an extended and selective bacterial death occurs following laser irradiation in a dose dependent manner. Therefore, the new findings might impel studies on these antibacterial nanomaterials and their biological and medical applications.

Drug-resistance crisis has become a global concern. High mortality rates associated with MRSA infections are the result of the increase in drug-resistance via natural selection^1^. The problem becomes even more serious in hospitalized patients who are at higher risk of bacterial infection. Drug-resistance has been shown to double the length of hospitalization, with a similar impact on mortality and morbidity, thus calling for extreme care when it comes to antimicrobial therapy in an attempt to reduce unnecessary antibiotic exposure^2^.

The negative impact of β-lactam and penicillin-resistant MRSA isolates regards both community-acquired and hospital-acquired infections^3^. In recent years, Methicillin-resistant *S. aureus* has become one of the most common pathogens that cause nosocomial infections, being endemic in most countries in Europe. It is very difficult to eradicate this bacterium from hospitals due to its genetic variation. Mutations acquired by individual bacteria or horizontal gene transfer lead to a decrease in the effectiveness of antibiotics and thus resistant traits proliferate^4^.

Despite recent development of new antibiotics, most of them are only variations to already existing drugs that show low efficiency in MRSA infections. There is need for novel technologies that would help detect and treat MRSA infections quickly^5^.

The importance of nanotechnology becomes relevant when it comes to drug delivery, which could be optimized by using targeted nanoparticles (NPs) as viable drug delivery systems^6^.

Recent progress in chemical functionalization has seriously influenced the development of novel nanomaterials, as well as their use in biological and medical applications. The flexibility displayed by the physical and chemical properties of gold nanoparticles (GNPs) and their strong absorption in the near-infrared region (NIR) make them suitable for a wide range of biomedical applications^7^.

Extensive studies on photothermal properties of GNPs conducted by various authors using transient absorption spectroscopy indicated the high absorption of gold nanostructures in the ultraviolet (UV) and visible range of detection, making them appropriate for applications in nanophotothermolysis experiments^8^.

Gold nanoparticles bound to various antibodies may exhibit target specificity for a key molecule from bacterial membrane. Once reach at the bacterial site these nanoparticles can be activated under NIR irradiation to transform photon energy into heat. This phenomenon would further lead to the disruption of bacterial membrane^9^.

This therapeutic approach has been recently exploited by many research groups^10^. In this context we have performed synthesis of gold nanoparticles (GNPs) by wet chemistry followed by bio-functionalization with IgG molecules. Following administration of IgG-GNPs to MRSA cultures at various concentrations and various incubation time laser irradiation was performed. The presented results indicate that following IgG-GNPs biomolecule administration in MRSA culture an extended and selective bacterial death occurs following laser irradiation in a dose dependent manner.

## Results

### Functionalization and characterization of Gold Nanoparticles

**Figure 1:**
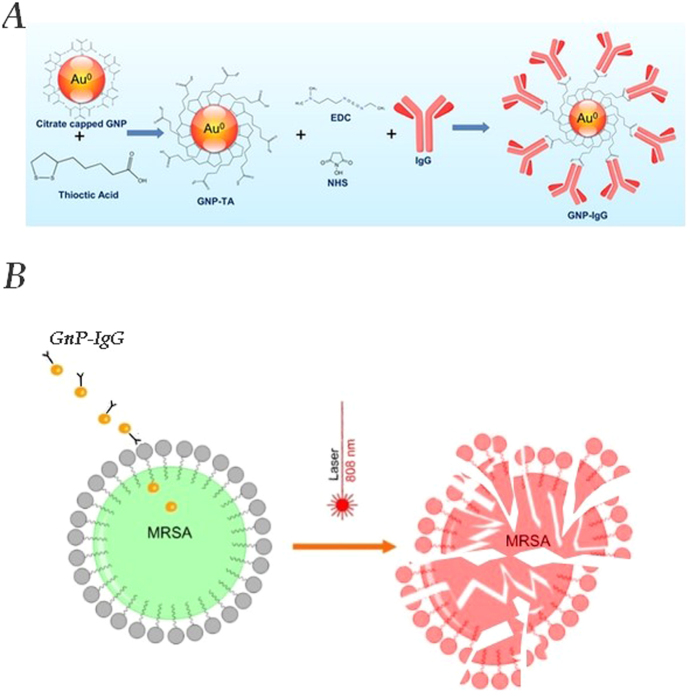
(**A**) Synthesis protocol illustration for the GNP-TA-IgG bio-nanocomposite (**B**) Schematic illustration of the proposed antimicrobial nanotherapy against MRSA, using laser IgG functionalised gold nanoparticles.

Citrate capped, thioctic acid capped and IgG functionalized gold nanoparticles (Fig. 1) were characterized by means of UV-Vis, FT-IR, DLS and AFM techniques.

**Figure 2:**
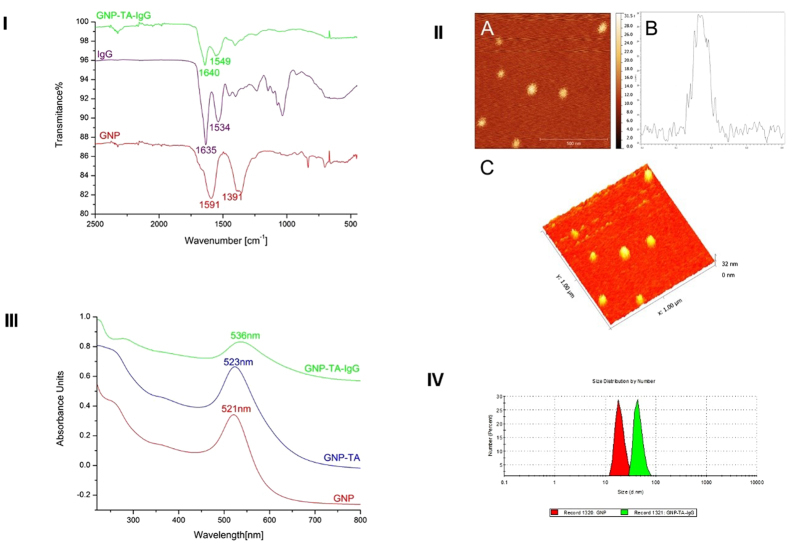
(**I**) FT-IR spectra of citrate capped GNPs, IgG and GNP-TA-IgG samples (region 2500-450 cm^−1^). AFM measurements of GNP-BSA: A. 2D image of IgG functionalized GNPs; B. Cross section graph of a single gold nanoparticle functionalized with IgG; C. 3D image of IgG functionalized GNPs. (**III**) UV-Vis spectra for GNP (red line), GNP-TA (blue line) and GNP-TA-IgG (green line) samples. (**IV**) DLS size distribution curves for the GNP (red) and GNP-TA-IgG (green) samples.

The UV-Vis spectra for the GNP, GNP-TA and GNP-TA-IgG samples is presented in Fig. 2III. The citrate capped gold nanoparticles present a surface plasmon resonance (SPR) peak at 521 nm^11^,^12^. In the case of the GNP-TA sample, the SPR peak is centred at 523 nm, this 2 nm bathochromic shift is attributed to the successful place-exchange reaction between the initial capping agent, citrate, and the thioctic acid. After the functionalization of GNP-TA with IgG another, bathochromic shift of 13 nm is registered in the UV-Vis spectra of the GNP-TA-IgG, this can be attributed to the bio-nanostructure increase in diameter due to the presence of the antibody on the gold nanoparticle surface.

To investigate the diameter size of the gold nanoparticles before and after functionalization with the IgG antibody DLS measurements were taken. The size distribution curves for the GNP and GNP-TA-IgG samples are shown in Fig. 2IV. 2, both samples presented themselves as monodisperse and stable at 20 °C. The increase in size, after the functionalization step, suggested by the UV-Vis spectra is confirmed by the DLS analysis. The hydrodynamic diameter for GNP is 17 nm while for the GNP-TA-IgG sample the registered diameter is 42 nm.

In order to confirm the successful functionalization of the gold nanoparticles with the IgG antibody, the IR spectra for citrate capped GNPs, IgG and the GNP-TA-IgG bio-nanocomposite were recorded. Figure 2I depicts the IR spectra in comparison for the 3 samples, in the region 2500-450 cm^−1^. The absorption peaks at 1591 and 1391 cm^−1^ registered in the case of citrate capped GNPs are attributed to the antisymmetric and symmetric stretching of COO^−^ of citrate ions^12^,^13^.

The IgG antibody presents two absorption bands located at 1635 and 1534 cm^−1^ attributed to the amide I (C=O stretching) and, respectively, amide II (C-N stretching and N-H bending vibrational modes). These amides I and amide II bands are also present in the GNP-TA-IgG bio-nanostructure at 1640 and 1549 cm^−1^ 14. Thus, for the GNP-TA-IgG sample, the disappearance of the citrate ions attributed IR bands, corroborated with the appearance of the IgG amide bands confirms the successful functionalization of gold nanoparticles with the antibody.

Atomic force microscopy measurements were conducted in order to investigate the size and shape of the synthetized GNP-TA-IgG bio-nanostructure. Figure 2II. A depicts a 2D representation of the GNP-TA-IgG, a 3D representation is shown in Fig. 2IIC and a cross section of a single functionalized nanoparticle is presented in Fig. 2IIB. The IgG functionalized gold nanoparticles presented themselves as spherical in shape and with a mean diameter of 32 nm. The DLS measurements placed the GNP-TA-IgG size at ~42 nm, this can be explained by the fact that the DLS technique provides a mean hydrodynamic diameter of GNPs surrounded by the IgG antibody and the solvation layers^15^.

### Fluorescence microscopy

**Figure 3:**
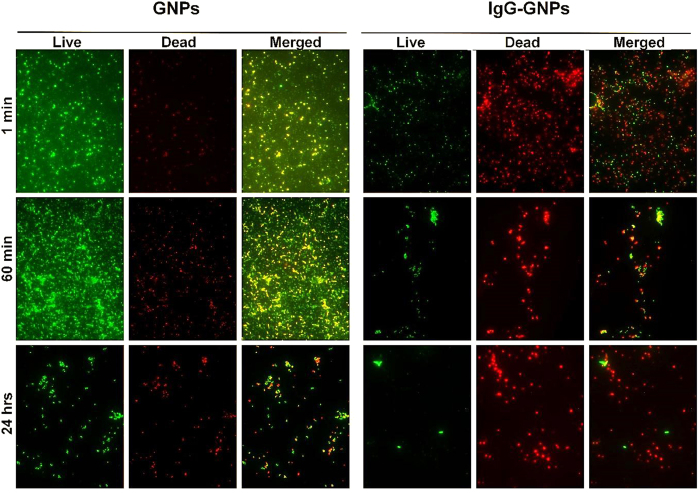
Viability of the bacterial population following treatment with 10 mg/L GNPs/IgG-GNPs at 1 min, 60 min and 24 hrs (LIVE/DEAD^®^ BacLight™ Bacterial Viability Kit) (red-dead bacteria, green-live bacteria). The merged images represent the superposed image of dead and live bacteria.

The extent of MRSA destruction after treatment was confirmed by fluorescence microscopy using the LIVE/DEAD BacLight kit. Figure 3 shows that the expression of propidium iodide (red channel) was significantly higher in Ig-GNPs-treated bacteria, thus indicating higher rates of death in MRSA cultures than in bacteria treated with GNPs alone (Chi square, p < 0.05).

As shown in Table 1, antibacterial activity against *S. aureus* was moderate to high for plasmon-activated GNPs, whose bacterial growth inhibition corresponded to 6.25% minimum inhibitory concentration (MIC) for the Ig-GNPs photothermal treatment. A 25% MIC was obtained for the solution containing GNPs alone.

### Flow cytometry

**Figure 4:**
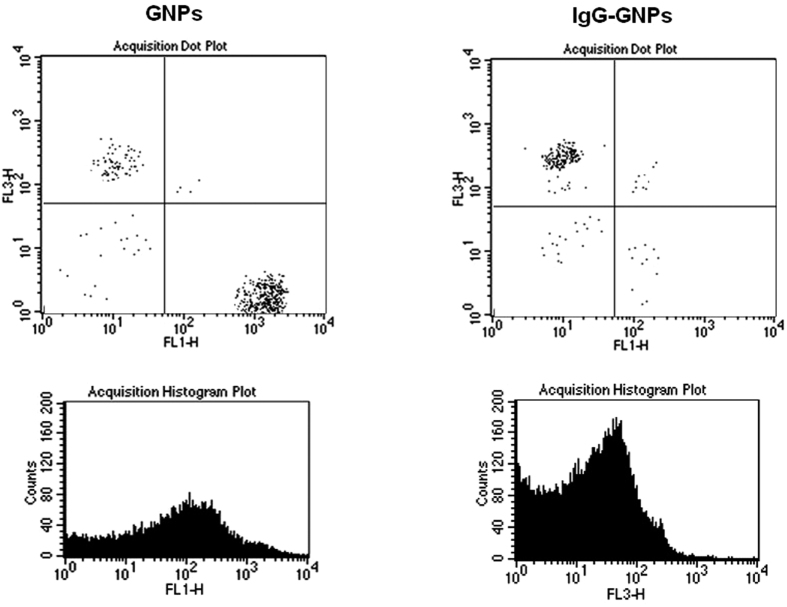
Flow cytometric quantification of the viability of MRSA bacteria following treatment with gold nanoparticles. Representative FACS dot plots gated on MRSA population are shown. Left upper panel shows less red fluorescence (FL3) suggesting high viability after GNPs administration, following incubation for 60 minutes with 10 mg/L. Right upper panel shows increased red fluorescence, strongly suggesting increased viability loss in IgG-GNPs treated MRSA bacteria. Histogram of red fluorescence (dead bacteria) in treated MRSA population following treatment IgG-GNPs (right lower panel) or live bacteria (green channel) in MRSA treated with GNPs (left lower panel). Proportions were calculated by expressing the number of events (bacteria) in the quadrant as percentage of the total number of cells. Quadrants (solid lines) were set using fluorescence dot plot generated by unlabelled MRSA alone.

**Figure 5:**
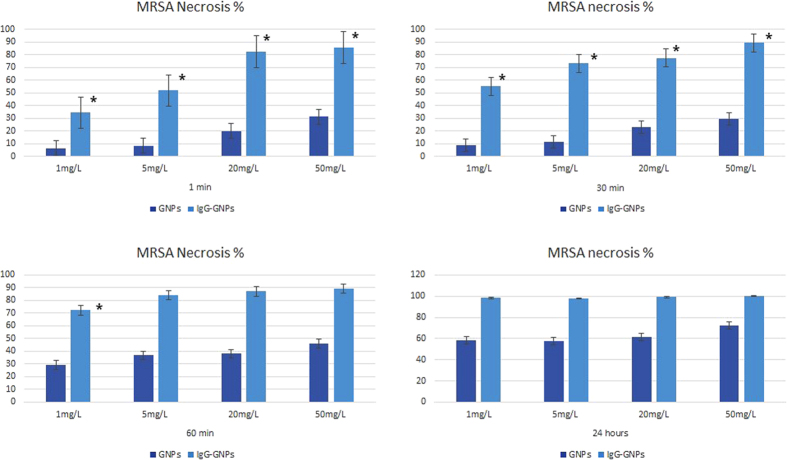
Results of experimental seriate exposure of MRSA to nanomaterials at different concentrations. Bars represent median dead cell percentage, as calculated by flow cytometry. Error bars represent range of dead cell percentage. MRSA treated with GNPs alone (control) for 60 sec and 30 min at concentrations ranging from 1 mg/L to 50 mg/L resulted in significantly lower necrosis rates. Following 24 hours treatment, the percentage of MRSA necrosis of the two GNP solutions in low concentrations (<20 mg/L) was of marginal statistical significance. Treatment with high concentration of GNPs for a long period of time (24 hours) was not significant in terms of necrosis percentage between the two MRSA cultures (GNPs and IgG-GNPs).

Local electronic structure changes dictate the antibacterial properties of nanoparticles following laser irradiation. Figures 4 and 5 show that GNPs killed MRSA at the rate of 28.4% (for 1 mg/L) and 72.6% (for 50 mg/L) in 60 seconds (p < 0.001), values that increased after 30 minutes, i.e. 45.2% (for 1 mg/L) and 88.2% (for 50 mg/L) respectively, p < 0.001.

The control group consisting of MRSA treated with GNPs alone for different time intervals (60 seconds, 30 minutes), at various concentrations (1 mg/L to 50 mg/L), showed significantly lower necrosis rates (8.3–39.6% for 60 sec, 9.8–51.3% for 30 min), (p < 0.001). Optimum MRSA destruction effect following treatment with IgG-GNPs was obtained for 5 mg/L concentrations and 30-minute time intervals (IgG-GNPs/GNPs: 53.3%/16.2%). After 24 hours’ incubation, the extent of MRSA destruction following treatment with low concentrations (<20 mg/L) of any of the GNP solutions was close to a marginal trend toward statistical significance (p = 0.081). High concentrations in the two treated solutions were associated with bacterial lysis, but with no significant differences between them (p = 0.316–20 mg/L; p = 0.438–50 mg/L) (Fig. 5).

### Image analysis

To further confirm flowcytometry results, ImageJ quantification of all fluorescent images following laser mediated treatment with GNPs-IgG (for exposure time varying from 1 min to 60 min) revealed a significantly increased red fluorescence suggesting strong induction of necrosis in MRSA population following the proposed treatment. (Chi square, p < 0.05).

## Discussion

Agents triggering physical bacterial destruction would be clinically essential for antimicrobial therapy in MRSA infections^16^,^17^,^18^,^19^,^20^.

The administration of potent active agents to the infection site enabling selective targeting of bacterial cells would thus represent the golden standard in MRSA treatment. They should also confirm drug efficacy. There is an urgent need for new site-targeted antibacterial agents, as antimicrobial resistance will still be an important issue in the following years. These alternative methods would eradicate bacteria and prove effective against all virulent strains^21^.

Antibiotic resistance occurs in different ways: molecular and epitopic changes on the surface of the microorganism, chromosomal mutations, enzymatic deactivation, outer cellular antibiotic transportation, periplasm^21^.

Due to their unique tunable properties gold nanoparticles were intensively explored in late years as laser directed nanoheaters since absorb light several million times greater in comparison with biomolecules. As result of their thermal activation temperature due to electron-phonon relaxation mechanism temperature rise significantly in the medium surrounding the nanoparticle resulting in local heating at a micrometric scale^10^. This phenomenon has been intensively explored by our group and others in the attempt to generate novel gold nanoparticle PT systems against multidrug resistant bacteria^17^. Gold nanoparticles were previously used for the selective nanophotothermolysis of the Sallmonella^22^.

Recently, several authors reported the functionalization of daptomycin- polydopamine-coated gold nanocages (AuNC@PDA) with anti-protein A antibody for the selective targeting Staphylococcus Aureus. This nanocompound showed high affininity for this type of bacteria and served as a multimodal therapeutic platform-photothermal and antibiotic^8^.

The obtained results presented in this paper were similar to other reports exploring the antibacterial potential of gold nanoparticles functionalized with antibodies as heat inducers agents under laser treatment^17^.

As suggested by some authors, the antibacterial action of GNPs occurs either by changing membrane potential and inhibiting ATP synthase in order to decrease ATP (with the activation of metabolic pathways generating anabolic reactions), or by inhibiting the ribosomal subunit for tRNA binding (restricting vital reactions in bacteria)^23^.

The rationale beyond this study was that abscess diagnosis coupled with ultrasound-guided drainage can be followed by this innovative approach. Delivery of functional moiety- attached gold nanoparticles associated with external laser irradiation could prove efficient in annihilation of MRSA biofilm and in preventing recurrence of infections. Moreover, maximal therapeutic benefit can be obtained while respecting a minimal level of invasiveness for the procedure (e.g. computer tomography/ultrasound- guided percutaneous or laparoscopic approach). We consequently dedicate our efforts to design a solution for the continuous need of novel targeting methods that would perform individual bacterial cell lysis associated with limited adverse effects.

Our previous reported data on safety of laser irradiation revealed no side effects *in vitro* when same parameters were used for laser exposure^24^,^25^,^26^. Several cell lines were used for testing. We therefore reason that presented treatment for complete extinction of MRSA intraabdominal/subcutaneous infections could be safely applied.

At the current time a serious concern of the scientific community is represented by the potential toxicity of nanomaterials with clinical applications on humans^27^. Abundant data has been reported on the toxicity of GNPs as fundament for potential medical applications of GNPs^28^. One very recent review offers a deeper insight on GNP safety testing^29^. There are several parameters that make interpretation of GNP toxicity data conflictual such as type of GNP, type of biomolecules coating the GNP, chemical and physical properties of GNPs (surface features: topography, area)^30^. While most of data sustain that GNPs are biocompatible and non-toxic some papers claim the opposite^31^. Future research is needed to elucidate the full spectrum of GNPs interactions in living systems.

We have developed an IgG gold nanoparticle based nanobiosystem that was tested on a multidrug resistant bacterium such as MRSA to demonstrate its selectivity and photothermal destruction capacity under NIR laser. The uniqueness of this finding might pave the way for new discoveries in nanotechnology-based antimicrobial treatment of infections.

However, there is need for more studies to assess unpredictable nanoparticle toxicities and biological interactions that might occur in biological systems.

## Methods

### Synthesis and characterization of Ig-GNPs

Sodium citrate, HAuCl_4_, thioctic acid (TA), sodium hydroxide, ethanol, sodium chloride, tri-sodium phosphate, N-(3-Dimethylaminopropyl)-N′-ethylcarbodiimide hydrochloride (EDC) and N-Hydroxysuccinimide (NHS) were all purchased from Sigma-Aldrich™ (Darmstadt, Germany). All reagents were used as received, without further purification.

The synthesis protocol for the IgG functionalized gold nanoparticles was comprised of three steps (Fig. 1A): first citrate capped gold nanoparticles (GNPs) were synthetized, the second step involved a place-exchange reaction between the citrate layer and thioctic acid in order to obtain thioctic acid capped GNPs and the third step which involved the covalent binding of the IgG antibody with the thioctic capped GNPs.

In order to obtain citrate capped gold nanoparticles a modified Turckevich method was employed. Briefly, 29 mg HAuCl_4_*3H_2_O were dissolved in 50 mL H_2_O bidist. and the solution was heated to 100 °C under continuous stirring. To this, a 5 mL sodium citrate solution (20 mg/mL) was quickly added and the reaction was allowed to continue for 2 h under reflux. Afterwards, the solution was allowed to cool down to room temperature. In the next step, thioctic acid was used in a place-exchange reaction in order to replace the citrate layer of the synthetized GNPs with thioctic acid. For this, the pH of a 10 mL citrate capped GNPs solution (~10 nM) was adjusted to 11 with a NaOH 1 M solution. Next, 100 μL TA (10 mM in ethanol) and 50 μL NaCl 2 M were added dropwise and under continuous stirring. The reaction was allowed to continue for 90 minutes at room temperature. Afterwards the obtained GNP-TA nanostructure to a centrifugation step (13200 RPM/20 min.) and redisspersed in ultrapure water.

For the functionalization of GNP-TA with IgG a covalent link was established between the carboxyl group of the thioctic acid and the amino groups from the antibody. First, the pH of a 2 mL IgG solution (5 mg/mL) was adjusted to 8.5 with the aid of Na_3_PO_4_ 0.1 M. To this, 500 μL EDC (30 mg/mL) and 500 μL NHS (30 mg/mL) were added and the reaction was perfected for 10 minutes. Next 2 mL of the GNP-TA solution, obtained in the previous step, were added and the sample was stirred vigorously for 10 min. at room temperature. Afterwards, the GNP-TA-IgG sample was subjected to a centrifugation step at 13200 RPM for 20 minutes and then re-dispersed in 10 mL ultrapure water. The obtained IgG functionalized GNPs were stable over several weeks at room temperature.

The UV-Vis spectral analysis was conducted on a Shimadzu UV-1800™ instrument. The UV-Vis spectra for the GNP, GNP-TA and GNP-TA-IgG samples were recorded 800 nm to 200 nm, with a spectral resolution of 0.5 nm. OriginLab^®^ software 7.0 was used to normalize all recorded spectra.

Dynamic light measurements (DLS) were performed on a Zetasizer – Nano S90 instrument (Malvern Instruments, Westborough, UK) at 37 °C at 90° scattering angle.

A Perkin-Elmer Spectrum Two^®^ instrument was used for the universal attenuated total reflectance fourier transform infrared spectroscopy (ATR-FT-IR) analysis. The instrument was equipped with an UATR single reflection diamond module. Baseline corrections and spectra processing were done using the Spectrum 10™ software.

Atomic force microscopy (AFM) measurements were taken with a Workshop TT-AFM^®^ (AFMWorkshop, CA, USA) operated in vibrating mode using ACTA-SS cantilevers (AppNano, CA, USA). The acquired AFM data was further processed with the Gwyddion^®^ 2.36 software.

### Nanoparticle therapeutics

In order to reach a 5–6*10^3^/ml density, MRSA bacteria were cultured in aqueous solution of amino acid (Sigma) in oxygen atmosphere. The solution was then added with plasmon-resonant GNPs followed by incubation (1 min, 30 min, 1, 5, and 24 h; 1, 5, 20, and 50 mg/L), centrifugation (10 minutes at 11,000 × g) and subsequent amino acid reimmersion. All experiments for all concentrations were carried out in triplicate.

### MRSA culture and characterization

After collecting an intraoperative swab sample from a surgical patient with intra-abdominal abscess, followed by MRSA agar inoculation and subsequent incubation (48 hours, 35 °C) in oxygen atmosphere, tube coagulase and DNase tests helped confirm the MRSA colony.

The cefoxitin disk screen test helped detect resistance in MRSA. Microorganisms resistant to all non-β-lactam antibiotics (tetracycline, ciprofloxacin, erythromycin, trimethoprim/sulfamethoxazole) were identified as multi-drug resistant.

The antibacterial effect of both types of GNPs was assessed using minimum inhibitory concentrations (MIC) employing the dilution method using 96-well microplates as previously described^32^.

Our experiments consisted of ~10^4^ CFU/mL bacterial concentration in order to determine the bactericidal effect of GNPs and IgG-GNPs. Baseline GNP/IgG-GNP dispersion was defined as minimum inhibitory concentration. Therefore, 50% MIC values indicate 50% nanoparticle content in the original synthesis. Culture medium evaporation was lowered during incubation by adding 200 μL deionized water on the inner microplate perimeter. From the culture medium, 100 μL were administered in 12 of the 96 wells, except for well no. 1, subsequently adding 100 μL GNP dispersion in the first and second wells. Following micropipetting, 100 μL were transferred from well no. 2 to well no. 3 and further on up to well no. 11. The wells were added with 10 μL MRSA solution (approx. 10^4^ CFU/mL), except for well no. 11, followed by 24 hours incubation at 37 °C in oxygen atmosphere.

From each well, 5 μL were inoculated in Petri dishes containing a growth medium to enable the assessment of the inhibitory effect of the nanoparticles. Inoculum density was set to give 10^4^ colony-forming units (CFU) per spot on the agar. The lowest MIC was defined for the absence of bacterial growth, disregarding a single colony within the surface of the inoculated spot. Positive (growth medium + inoculum) and negative (growth medium + antimicrobial) controls were represented by wells no. 11 and 12.

To indicate their possible inhibition against MRSA, GNPs underwent the same procedure.

### Viability measurements

After treatment, 1 × 10^4^ microorganisms in MEM non-essential amino acid solution were pelleted by centrifugation (14,000 RPM, 30 minutes). The LIVE/DEAD^®^ BacLight™ Bacterial Viability Kit (Invitrogen) was used to assess MRSA viability after treatment.

### Flow cytometry analysis

Green (collected in the FL1 channel −530 nm) and red (collected in the FL3 channel >670 nm) fluorescence based assays for MRSA viability using the BD FACSCalibur flow cytometer (Becton Dickinson, San Jose, CA, USA) were used to differentiate between live and dead microorganism populations. The LIVE/DEAD BacLight kit was employed to assess bacterial cultures after treatment with IgG-GNPs. Flow cytometry was used to analyze MRSA suspensions following treatment with GNPs or IgG-GNPs as indicated by the manufacturer. The resulting histogram and Cell Quest pro software helped determine the amount of live and dead bacteria. Data were acquired at low speed and in logarithmic mode (approx. 15 μl min^−1^) to keep count below 1,000 events s^−1^. Bacterial populations were enumerated using the Bacteria Counting Kit for flow cytometry including the SYTO^®^ BC green fluorescent dye that easily penetrates gram-negative bacteria. The green fluorescence channel gaining signals from fluorescent bacteria displayed data in the forward scatter versus side scatter plot, as indicated by the manufacturer.

### Laser irradiation of gold nanoparticles

Photoexcitation of gold nanoparticles (1 ml of solution containing different concentrations of GNPs placed in an ~1cm^2^-bottom glass cuvette) was performed for 10 minutes with a high power (up to 2 W/cm^2^) 808 nm diode laser fixed in vertical position about 2 cm above the surface.

### The MRSA-Screen latex agglutination test

The test was performed to rapidly detect PBP2′, being of great usefulness for accurate MRSA detection. Five μl of MRSA suspended in 0.1 M NaOH underwent boiling for 3 minutes, subsequently adding 50 μl of 0.5 M KH2PO4. Following centrifugation (1,500 × g, 5 minutes, room temperature), 50 μl of supernatant was mixed with the latex reagent on a test card and 50 μl of supernatant was mixed with 25 μl of negative-control latex. After being mixed on a shaker, PBP2 could be detected by agglutination within 3 minutes^33^.

### ImageJ analysis

For the quantitative analysis of red, necrotic bacteria following IgG-GNPs mediated treatment, fluorescence quantification was done using ImageJ (National Institutes of Health, New York, NY, USA) software by measuring the stained area on a similar number of microorganisms as previously described^25^. Values are expressed as the percentage of the red intensity in bacterial population compared with control cells.

### Statistical analysis

Data were expressed as mean and standard deviation. The normality of data was assessed using the Kolmogorov-Smirnov test and dependent samples were compared using the Wilcoxon signed-rank test. The significance level was below 0.05 in all situations. Microsoft Office Excel Application and SPSS Statistics 17.0 were used for data processing.

### Ethical Consideration

The authors have obtained the institutional board approval (decision number 7699 issued by an Independent Research Ethics Board from Regional Institute of Gastroenterology and Hepatology Octavian Fodor Cluj) for the experiments outlined in this paper and have followed the principles outlined in the Declaration of Helsinki. Informed consent has been obtained from the participants involved, and the experiments were constructed in such manner that would not affect the future therapeutic approach in these patients.

Human and animal rights compliance statement. All procedures outlined in this paper were performed in conformity with the ethical standards of the responsible committee on human experimentation (institutional and national) and with the Helsinki Declaration of 1975, as revised in 2008.

## Additional Information

**How to cite this article:** Mocan, L. *et al*. Selective *in vitro* photothermal nano-therapy of MRSA infections mediated by IgG conjugated gold nanoparticles. *Sci. Rep.* **6**, 39466; doi: 10.1038/srep39466 (2016).

**Publisher's note:** Springer Nature remains neutral with regard to jurisdictional claims in published maps and institutional affiliations.

## Figures and Tables

**Table 1:** Minimum Inhibitory Concentration (MIC) of gold nanoparticles against MRSA.

Wells	%	GNps	Pre-Gnps
1	100	−	−
2	50	−	−
3	25	−	−
4	12.5	+	−
5	6.25	+	−
6	3.13	+	+
7	1.56	+	+
8	0.78	+	+
9	0.39	+	+
10	0.20	+	+
11	0.10	−	−
12	−	+	+

(+)-growth of bacteria. (−) no growth of bacteria. 11-negative control/12 positive control.
